# Temperature stress deteriorates bed bug (*Cimex lectularius*) populations through decreased survival, fecundity and offspring success

**DOI:** 10.1371/journal.pone.0193788

**Published:** 2018-03-14

**Authors:** Bjørn Arne Rukke, Ranjeni Sivasubramaniam, Tone Birkemoe, Anders Aak

**Affiliations:** 1 Department of Pest Control, Norwegian Institute of Public Health, Oslo, Norway; 2 Faculty of Environmental Sciences and Natural Resource Management, Norwegian University of Life Sciences, Ås, Norway; Texas A&M University College Station, UNITED STATES

## Abstract

Sublethal heat stress may weaken bed bug infestations to potentially ease control. In the present study, experimental populations exposed to 34, 36 or 38°C for 2 or 3 weeks suffered significant mortality during exposure. Among survivors, egg production, egg hatching, moulting success and offspring proliferation decreased significantly in the subsequent 7 week recovery period at 22°C. The overall population success was negatively impacted by increasing temperature and duration of the stress. Such heat stress is inadequate as a single tool for eradication, but may be included as a low cost part of an integrated pest management protocol. Depending on the time available and infestation conditions, the success of some treatments can improve if sublethal heat is implemented prior to the onset of more conventional pest control measures.

## Introduction

Bed bugs (*Cimex lectularius*) have great success in urban areas and encounter few constraints in their environment. With their current global distribution, human dwellings may even be considered their main “natural” habitat, and bed bugs are a true anthropochore. Food through human blood meals is available in sufficient quantities, cryptic nocturnal behaviour enables undisturbed feeding [1, 2], aggregation in cracks and crevices offers suitable microhabitat [3], hitchhiking on human belongings [4, 5] or walking [6, 7] secures efficient dispersal, natural enemies [8–10] are mostly absent, the high reproductive rate promotes fast population growth [11, 12] and resistance counters our attempts to use pesticides for elimination [13–17]. Many of these success factors are difficult to manipulate in bed bug control, but the abiotic environment in which bed bugs thrive is disposed to alterations. In natural ecosystems, even minor increases above optimal habitat temperature can have a major effect on insect success [18–21] through altered life history parameters that decrease fitness and ultimately survival. Human sleeping quarters are usually confined spaces where temperature in well-insulated buildings can be adjusted by 15–20°C using heaters and air conditioners. This could be useful in bed bug control by ensuring stressful conditions above their preferred temperature range that ends at 32°C [22].

Managing bed bugs within a community requires thorough protocols that involve all relevant stakeholders, such as tenants, managers and pest controllers, and should include preventive measures, persistent monitoring for early detection, efficient containment of dispersal and a public awareness of basic bed bug biology [2, 23, 24]. As introductions will still occur, there is no way around an eradication strategy that requires a combination of several killing agents, such as desiccant dusts [25–28], regular chemical pesticides [2, 29, 30], vacuuming [30], trapping [31, 32], bio-pesticides [8–10], steam treatment [33] and cold [34–36] or heat treatment [37–39]. Such Integrated Pest Management (IPM) is necessary, because it is unlikely that a new single method will make bed bug control easy again, and consequently focus must be directed towards improving existing control techniques and combinations between them. Heat stress may potentially benefit control [40], and the response is dependent on temperature and treatment duration. All bed bugs die within a few minutes at 60°C [41], within an hour at 50°C [22, 39], in 2 days at 40°C and in 9 days at 38.5°C [40]. Some bed bugs will die if exposed to lower temperatures for extended periods, but more interesting are the sublethal effects on fecundity and offspring success that have been observed at temperatures as low as 34.0°C [40]. All heat exposure below 38°C and above favourable conditions that end at 32°C [22], can be defined as sublethal heat stress for bed bugs because individual performance will decrease without subsequent rapid death. If circumstances allow constant or periodic infliction of temperature stress, it may put constraints on the bed bug population prior to conventional control. The effects of heat stress on insects in general depend on intricate interactions between exposure time, temperature stability, acclimation, species adaptation and physiological protective abilities. The consequences of heat stress are subtle and often the sum of many factors acting together within a sublethal range [42, 43]. Population performance sums up the effects across life stages and is clearly the most relevant measure when relating temperature stress to bed bug pest control.

Several physiological mechanisms govern the response to heat stress. Factors, such as denaturation of proteins, accumulation of toxic products, DNA damage, pH changes, loss of membrane function, nutrient deprivation and desiccation [43, 44], will harm insects directly, but they may also be affected indirectly through shifts in microbial infection rates or disruption of symbiotic relationships. Heat-challenged insects that develop a disrupted symbiont system often exhibit retarded growth, increased mortality, reduced fertility, abnormal body colour and other defective phenotypes [21, 45–47]. The bed bug *Wolbachia*-symbiont [48] may consequently be a target when attempting to induce detrimental effects through sublethal stress. If this stress is handled poorly by the symbiont, its provision of essential nutrients will be affected, which will reduce survival, development and fecundity of the bed bugs [49, 50]. Activation of protective heat shock proteins, which counter the negative consequences of thermal stress, has been observed in bed bugs after heat exposure [36] and blood feeding [51], and bed bugs are known to recover partially under normalized temperatures [40]. Therefore, it is important to evaluate the consequences of heat stress in response to different temperature regimens that reflect potential control situations, and performance should be evaluated for extended periods to assess potential recovery.

The lower limits for observable adverse effects of heat stress are incompletely described for bed bugs, and the consequences of extended duration and alternating stress-recovery have not been investigated. We aimed to fill this knowledge gap, and in the present study the effects of prolonged constant and disrupted sublethal heat stress were examined at temperatures of 34–38°C for 2 and 3 weeks. Survival, fecundity and offspring success were evaluated during and after heat stress to help describe short- and long-term population effects and connect these observations to IPM-aspects of bed bug control.

## Materials and methods

### Bed bug stock cultures

Stock cultures originated from bed bugs collected at two locations in Oslo, Norway in 2009. The bed bugs were fed on heated human blood through a Parafilm membrane [52]. Two of the authors donated small samples of blood voluntary, after written consent (S1 File). Approval from an institutional review board or ethics committee was not required. They were maintained on a photoperiod 16:8 (L:D) h at 22°C and 60% relative humidity (RH) in climate chambers (Sanyo MLR-351H; Medinor ASA, Oslo, Norway).

### Experimental units

All experimental bed bugs were kept on filter paper (47 mm qualitative filter paper; VWR, Oslo, Norway) in polyethylene boxes (140 mL straight sample container; VWR). The lid had a circular 40 mm diameter hole with an embedded metal mesh screen (0.25 mm opening; Burmeister AS, Oslo, Norway) allowing air passage and feeding. To ease handling and registration, experimental units started with three male and three female adults as well as six fifth instar nymphs, except in the offspring development part of the study, which started with six first instar nymphs in each population box. Development of different stages in each box was allowed during the experiments to mimic potentially rebounding populations in a field situation.

### Sublethal heat-exposure experiment

Fourth and fifth instar nymphs were collected from the stock cultures to establish fresh colonies. They were fed and left for 2 weeks to allow moulting. Only newly emerged individuals were used. All adults were fed 2 days prior to the onset of heat stress. Nymphs were not given access to blood at this stage to avoid moulting into adults during heat stress. ***Heat stress*** was applied by keeping the populations in the climate chambers with 60% RH and temperatures of 34, 36 or 38°C. These temperatures were chosen as they represent a thermal regimen in which sublethal effects after prolonged exposure likely occur in bed bugs [40, 53]. The bed bugs were either kept at these temperatures throughout the experiment (constant treatment), or in periods of 3 days intermixed with 4 days of “rest” at 22°C (disrupted treatment) for 2 or 3 weeks. The 2-week treated bugs spent the third week at 22°C and 60% RH. We used 10 experimental units with a total of 60 adults and 60 nymphs for each of these 12 heat stress-treatments (1,440 bed bugs in total) and 10 control units with a total of 60 adults and 60 nymphs kept at a constant 22°C and 60% RH. Mortality was checked daily during the heat stress period by blowing gently through the ventilated lid and observing the behavioural responses. Bed bugs laying on their back not moving legs and antennas when gently touched with tweezers, were considered dead. Only dead individuals were handled. Egg production and egg viability were recorded at the end of this 3-week period. To investigate ***population recovery*** by measuring the populations’ ability to grow at 22°C after experiencing thermal stress, the survivors from each treatment were reorganized into new boxes to make as many complete experimental units with three females, three males and six fifth instar nymphs as possible. These bed bugs were fed four times 14 days apart during a 7-week period. Two days after the last feeding, the numbers of eggs and nymphs, nymph stage and the new total number of adults were recorded. At the same time, we transferred offspring from these recuperated populations by selecting 30 newly emerged first instar nymphs from each treatment and distributed them into five new boxes. The nymphs in this ***long-term effect experiment*** were fed three times, 14 days apart, over a 6-week period, and the developmental stage reached by the nymphs was recorded 2 weeks after the last feeding.

### Statistical analysis

All data were analysed using SigmaPlot 13.0 (Systat Software Inc. Son Jose, CA, USA) and JMP pro 13.0.0 (SAS Institute, Cary, NC, USA) software. Multiple comparisons were performed using one- or two-way analysis of variance (ANOVA). The level of significance was set to 0.05. Dunnett’s method was used for multiple comparisons versus a control group. If the data were not normally distributed, we used the nonparametric alternative Kruskal–Wallis ANOVA. The Kaplan–Meier product limit method with the log-rank test between groups was used for survival analyses, where a population’s fate was investigated by following the 60 adults and 60 nymphs in each treatment for 21 days, and the time of death of each individual was registered on a daily basis. Adult and nymph survival data during heat stress were pooled because we wanted to investigate total population performance at the different time-temperature regimens. Egg production, hatching success and moulting data were pooled across 2- and 3-week treatments as there was no significant difference between these treatment lengths. We used a simplified population index to investigate the later population recovery after heat stress because the demographic measures needed to forecast population growth (age- or stage-specific schedules for reproduction, moulting and mortality [54]) could not be obtained by the experimental design. This index (***pi***) multiplies the number of nymphs (***n***) produced per female, the maximum stage (***s***) reached by these offspring and the number of new adults (***a***) developing from the original fifth instars. It was used for the relative comparison between treatments only and indicates population recovery potential by putting weight on offspring production at the same time as it increases the score if juveniles progress towards adulthood and if new adults emerges. To avoid zeroes in the equation, we added one to all numbers (population index (***pi***) = (***n*** + 1) × (***s*** + 1) × (***a*** + 1)). First instar nymphs produced by adults that had previously been maintained at 34°C for 2 weeks were mistakenly killed leading to one missing treatment in the long-term effect experiment.

## Results

### Mortality and reproduction under heat stress

No mortality was observed in the control treatments, indicating limited impact from handling, rearing boxes, filter paper, artificial feeding or climate chambers at 22°C and 60% RH.

**Fig 1:**
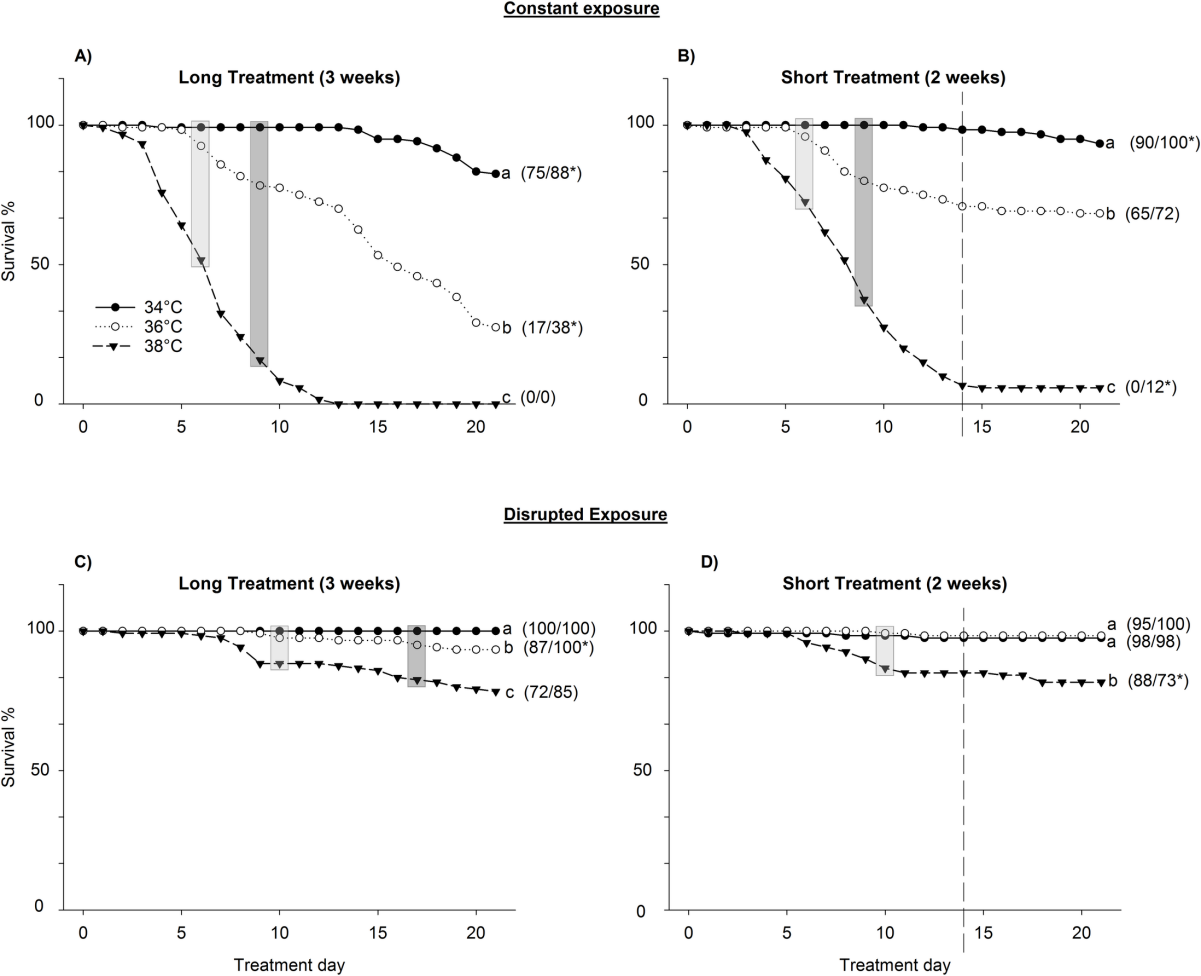
Survival of *Cimex lectularius* during heat stress. Survival during long (3 weeks) or short (2 weeks) heat stress in constant or disrupted temperatures. Numbers in brackets show percentage final survival of nymphs (left) and adults (right) in each treatment on day 21. * denotes significant difference in survival between adults and nymphs during the whole heat stress period (Kaplan–Meier survival analysis, p < 0.05). Otherwise, the data of nymphs and adults were pooled. The dashed vertical line indicates where thermal stress was terminated in the short treatments. The total treatment period was 21 days, and bed bugs in the short treatments spent the last 7 days at 22°C. Different letters (a, b, c, and d) denote significant differences (Kaplan–Meier survival analysis, p < 0.05) in survival between different heat treatments within each length-long/short combination. Rectangles indicate differences in mortality after experiencing a total of 6 (light grey) and 9 (dark grey) days at high temperatures.

In the heat treatments, the nymphs suffered higher mortality than the fed adults in five of the treatments but lower mortality in one treatment (Fig 1). Population mortality always increased with increasing temperature under constant heat stress (Kaplan–Meier, all pairwise comparisons Χ^*2*^ > 27.94, df = 1, p < 0.001, Fig 1A and 1B) and 3 weeks of exposure always induced higher mortality than 2 weeks of exposure at the same temperature (Kaplan–Meier, all pairwise comparisons Χ2 > 8.61, df = 1, p < 0.004). The disrupted 3-week treatments also showed a temperature dependent increase in mortality (Kaplan–Meier, all pairwise comparisons Χ^*2*^ >8.24, df = 1, p < 0.005, Fig 1C), whereas the 2-week treatments only experienced significant mortality at 38°C (Kaplan–Meier, pairwise comparisons at 34°C and 36–38°C, Χ^*2*^ >15.94, df = 1, p < 0.001, Fig 1D). Disrupted treatments at the same temperatures were not influenced by treatment length (Kaplan–Meier, all pairwise comparisons of 2 vs. 3 weeks Χ^*2*^ < 3.71, df = 1, p > 0.05).

**Fig 2:**
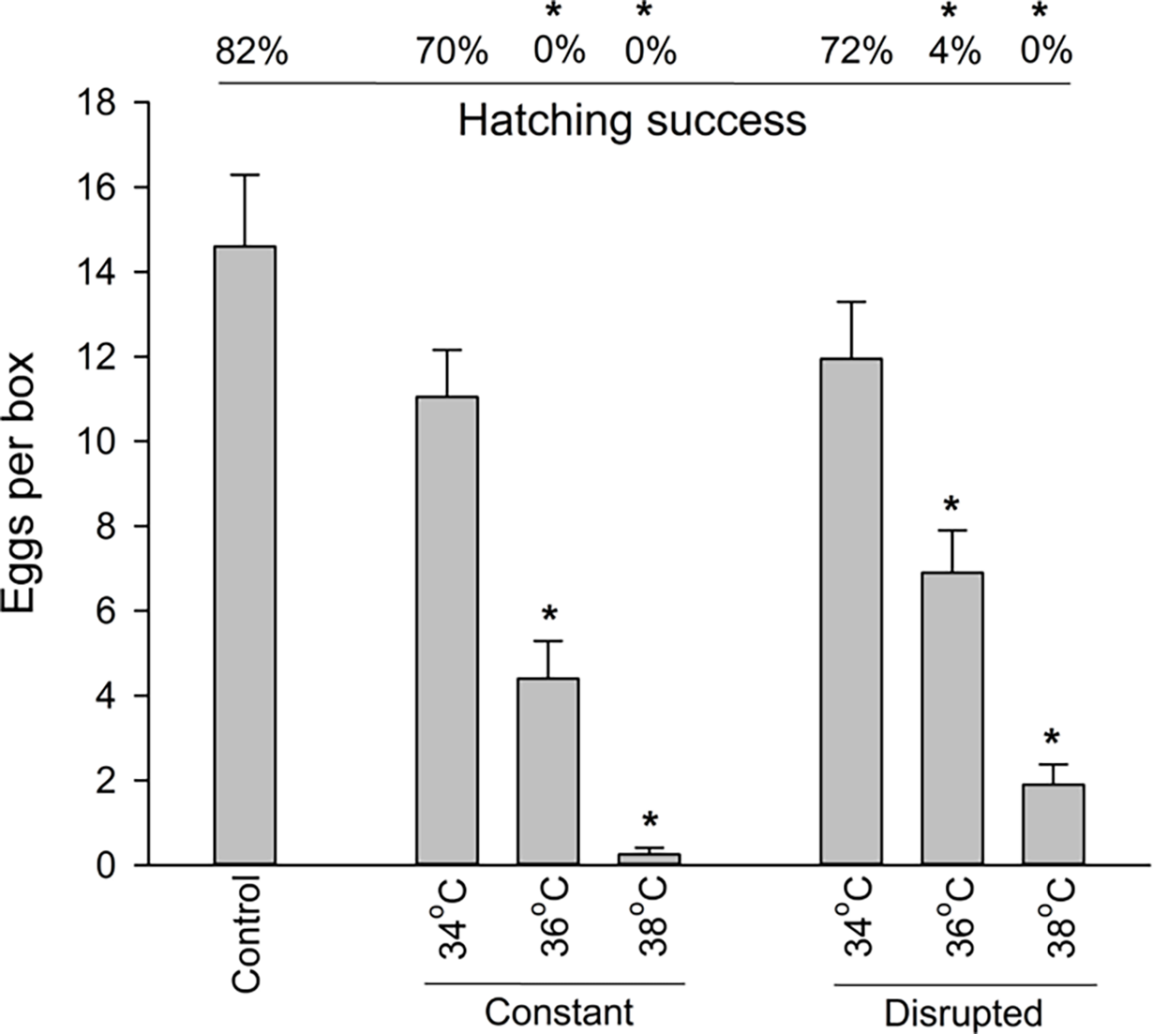
Egg production and hatching success of *Cimex lectularius* during heat stress. Egg production (mean number of eggs per box) and hatching success (nymphs/eggs in percent) in populations during the constant and disrupted heat treatments. Data from the 2 and 3 week treatments are pooled. * denotes significant difference relative to control (Dunnett’s method, p < 0.05).

Egg production and hatching success decreased with increasing temperature in both the constant and disrupted temperature regimens, whereas no difference was observed between 2 and 3 weeks of exposure (Table 1 and Fig 2). The numbers of deposited eggs in the 36°C and 38°C disrupted treatments were reduced to 47% and 13% of the control respectively, and corresponding constant treatments were 30% and 2%. Hatching success at these four temperature regimens was < 5%.

**Table 1:** Effects of heat stress on *Cimex lectularius* population development.

		During heat				After heat			
		**Adults**							
**Adults**		Egg production		Egg hatching		Egg production		Egg hatching	
		Constant	Disrupted	Constant	Disrupted	Constant	Disrupted	Constant	Disrupted
	Temp	**<0.001**	**<0.001**	**<0.001**	**<0.001**	**0.048**	0.229	**<0.001**	**<0.001**
	Length	0.568	0.886	0.209	0.800	0.066	0.445	**<0.001**	**0.009**
	T × L	0.963	0.262	0.249	0.978	0.345	0.502	**0.010**	**0.031**
		**Nymphs**							
**Nymphs**		Adult emergence		Offspring moulting		Adult emergence		Offspring moulting	
		Constant	Disrupted	Constant	Disrupted	Constant	Disrupted	Constant	Disrupted
	Temp					*One-way ANOVA*, *p = 0*.*024*	**<0.001**	*One-way*	**0.002**
	Length	*No data*	*No data*	*No data*	*No data*		0.409	*ANOVA*,	0.785
	T × L						0.785	*p = 0*.*068*	0.992

Analyses of variance (ANOVAs) testing the effect of temperature (34, 36 or 38°C), exposure length (2 or 3 weeks) and the interaction between these two variables on egg production, egg hatching rate, adult emergence (fifth instar nymphs developing into adults) and offspring moulting success. The different ANOVAs show separate results of the disrupted and constant treatments during or after the heat stress of *Cimex lectularius*. Only p-values are given. The unbalanced heat stress result only allowed the use of a one-way ANOVA for adult emergence and offspring moulting after the constant treatments.

### Population recovery after heat stress

**Fig 3:**
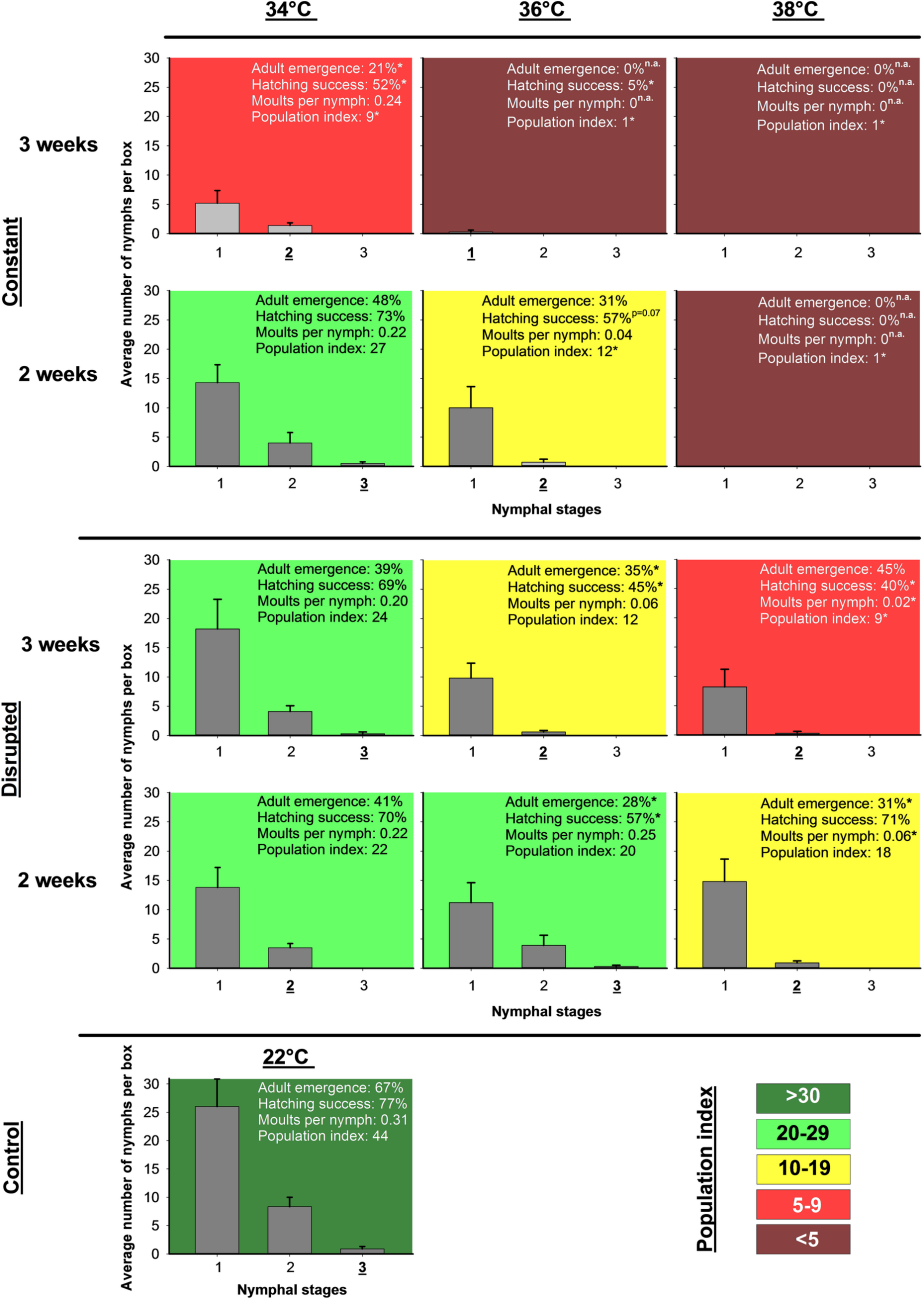
Population development of *Cimex lectularius* after heat stress. Population development in the 7 weeks after the different heat treatments in boxes that originally contained three females, three males and six fifth instar nymphs. All subplots show the number of nymphs produced in different stages with the highest development stage reached underscored. Given as text are **adult emergence** (percentage of fifth instar nymphs developing into adults), egg **hatching success**, number of **molts per nymph** and the **population index** ((number of nymphs produced per female + 1) × (maximum nymphal stage reached + 1) × (number of new adults developing from fifth instar nymphs + 1)). The population index is further visualised by the background colour of the subplots. The 38°C constant treatments are marked as not applicable (n.a.) because all the adults died during heat stress. * denotes significant difference relative to control (Dunnett’s method, p < 0.05).

**Fig 4:**
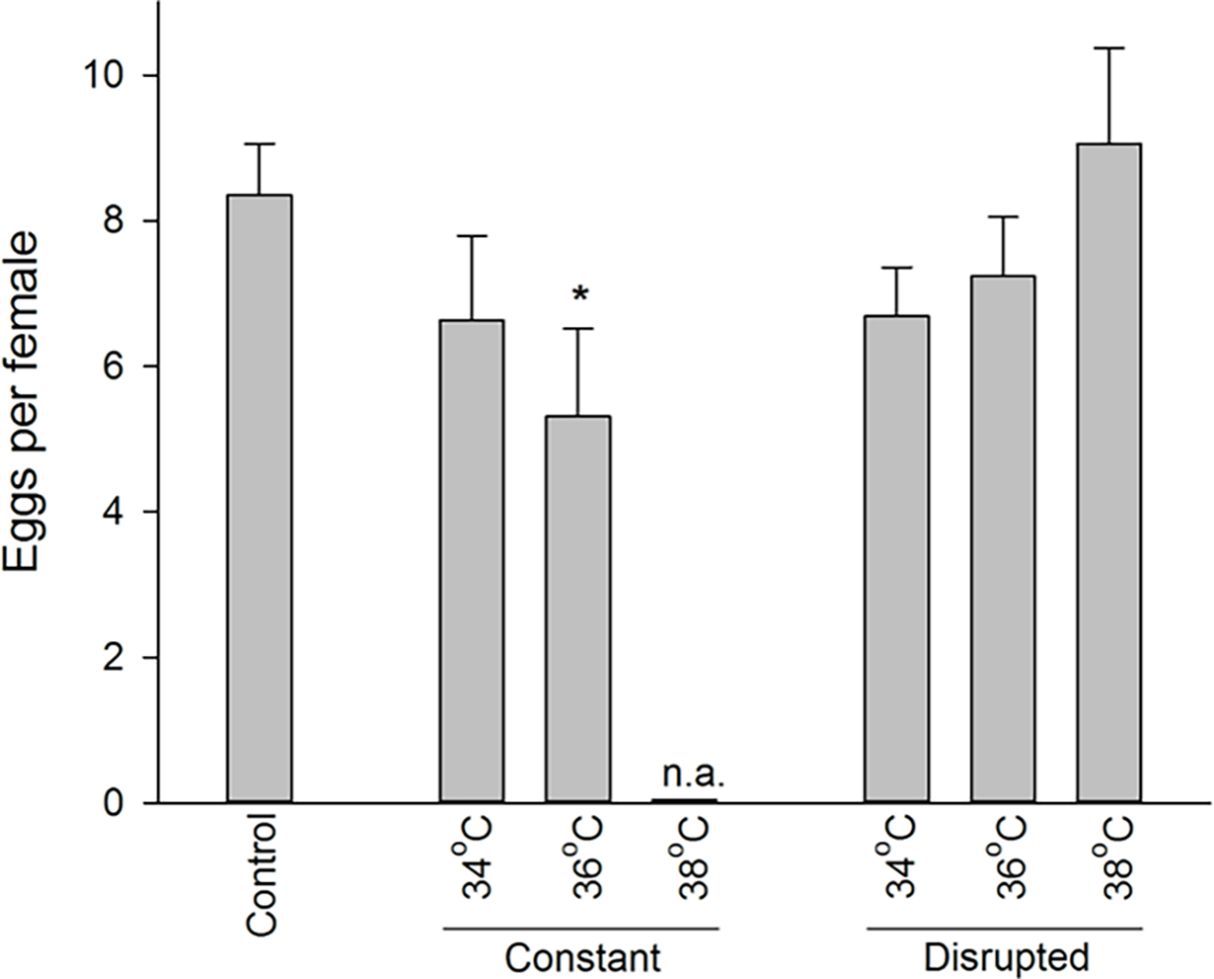
Egg production of *Cimex lectularius* after heat stress. Egg production in *Cimex lectularius* populations (mean number of eggs per female) after 7 weeks of the constant and disrupted heat treatments per female. Data from the 2 and 3 week treatments are pooled. * denotes significant difference relative to control (Dunnett’s method, p < 0.05).

The most severe heat treatments in the parental generation, both the 38°C constant treatment and 36°C constant 3-week treatment, had a population index of 1, suggesting heavily decimated bed bug populations (Fig 3, dark red). Less intense heat stress resulted in significantly restricted future population development closely connected to stress intensity (Kruskal–Wallis comparing the population index (pi) of the different treatments: H = 64.887, df = 12, p < 0.001, Fig 3; from lower left to upper right), even though no further mortality was observed among the survivors. The joint contributors to this overall effect were moulting, fecundity and offspring success. First, we observed a reduced ability of fifth instar nymphs to moult into adults (Table 1). This adult emergence decreased strongly with increasing constant heat stress, whereas an approximate 50% reduction was observed in the disrupted stress treatments compared to the control across all treatments (Fig 3). Second, populations that survived 36°C constant heat stress produced fewer eggs than the control, whereas no such decrease was observed following the 34°C treatments or any of the disrupted heat treatments (Table 1 and Fig 4). Third, both length and temperature affected the hatching success of eggs deposited by previously heat stressed adults, and a significant interaction was detected between the two factors (Table 1, Fig 3). The constant 2- and 3-week treatments and the disrupted 3-week treatments resulted in decreased egg viability as temperature increased compared to the control. Finally, the number of moults per nymph produced after heat stress decreased in the disrupted 38°C treatments. (Table 1, Fig 3). No nymphs molted in the 36°C 3-week constant treatment, and no molts were possible without surviving adults at the constant 38°C treatment.

### Long-term effect

**Fig 5:**
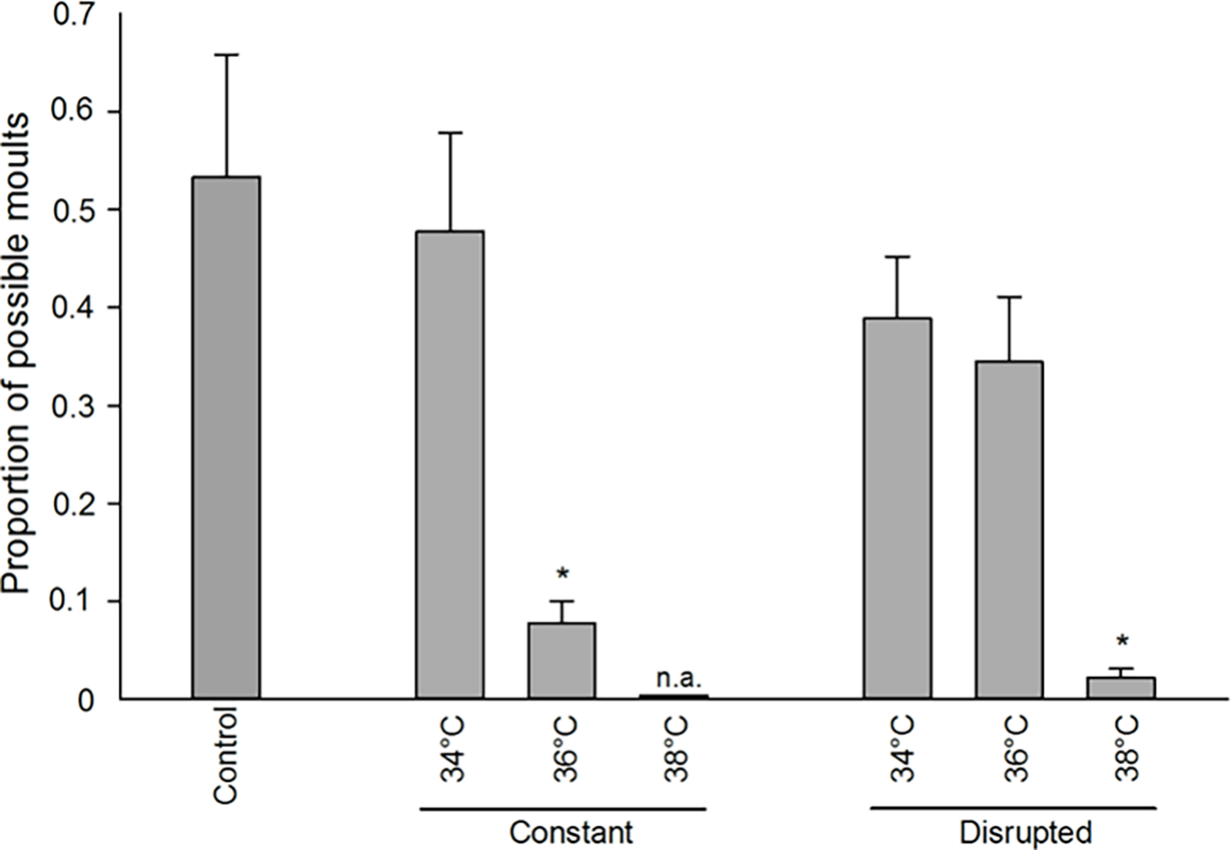
Long-term heat stress effects in *Cimex lectularius*. Development (proportion of possible moults per nymph) of *Cimex lectularius* first instar nymphs produced by adults that had been heat treated 7 weeks earlier. The nymphs were fed every 14 days and kept at 22°C for a total of 8 weeks. Each treatment is represented by five boxes with six nymphs each. Data from the 2 and 3 week treatments are pooled, except for the constant 34°C for which only 3 week data were available. No nymphs were produced at a constant 38°C (n.a.). * denotes significant difference relative to control (Dunnett’s method, p < 0.05).

First instar nymphs that hatched after 7 weeks of population recovery continued to show a reduced ability to develop into later instars (constant treatment: ANOVA; F_2,12_ = 7.080, p = 0.009, disrupted treatments: S1 Table, Fig 5). These long-term effects were present after constant 36°C heat stress and disrupted 38°C heat stress, and moulting was reduced to < 15% of the control.

## Discussion

The present study provides new knowledge regarding the effect from long lasting, sub-lethal heat stress on bed bug populations. The experiments reveal that extended exposure to sublethal temperatures causes deleterious effects in bed bug populations. A constant 3 week exposure to 34°C appears to be close to the limit for measurable heat induced mortality. Substantial mortality was observed during the stress period at the higher temperatures and also a considerable reduction in future recruitment was observed among survivors when temperatures were normalized. These results illustrate that sublethal heat stress could be applied as a tool in control efforts by reducing the growth potential of bed bug populations.

In most insects, elevated temperatures can be handled for short periods, but they become hazardous if exposure is prolonged [43, 55, 56]. Our results follow these general predictions and supplement previous reports of bed bugs maintained at comparable temperatures but shorter exposure durations [40]. We also observed substantially reduced mortality in the disrupted treatments compared to that in the constant treatments. This may be partly explained by fewer days at high temperatures accompanied by a reduction in physiological distress factors, but also by a reversal of negative processes or repair mechanisms [43, 44]. The fact that mortality after 6 and 9 accumulated days of disrupted heat stress was clearly lower compared to that after 6 and 9 days of constant stress (grey highlights in Fig 1) indicates that intermittent periods of relief from heat stress reduced the total effect of the experienced thermal exposure. Physiological heat resilience is incompletely understood in bed bugs, but they can at least upregulate heat shock proteins [36] to prevent protein denaturation and allow proteins to refold [57, 58].

The reduced fecundity observed during heat stress continued after the bed bugs were transferred back to 22°C and consequently decreased the overall reproductive value of the survivors. This is beneficial for controlling these pests, as fewer eggs, lower egg hatching success and deficient nymph development may lead to changes in the age- or stage-distribution followed by a deteriorated population growth potential [54]. The lack of normal offspring development in bed bugs several months after parental heat stress may be linked to the well-being of their *Wolbachia* symbionts that supply them with essential B vitamins [49, 59]. Sublethal heat can be used to remove *Wolbachia* infections from insects [60–62], and more specifically render the bed bug mycetomes symbiont free [53]. As *Wolbachia* is transmitted from mother to nymphs through the mycetomes during oogenesis [49], the offspring effects suggest that seriously heat stressed mothers may leave their progeny symbiont-free or at least heavily decimated.

The explanatory mechanisms behind the sustained population effects were not investigated in this study, but regardless if the observations originated from physiological injury, microbial disturbance or both, the indirect effects appear systematic and connected to both treatment length and temperature (population success index, Fig 3) in the same way as for mortality during treatment. This finding demonstrates the need for studies of the long-term effects from sublethal stressors, and the complexity of the mechanisms is further highlighted by the different responses at both ends of the temperature scale. Sublethal cold exposure affects adult bed bugs, but this is not sustained in their later offspring [35], whereas heat enforces both direct and cross-generational effects [40] to cause a prolonged detrimental population impact. These differentiated effects may be a further indication of *Wolbachia* regulated responses to heat stress.

Thermal stress may increase water loss, and desiccation is thus likely to act together with heat damage to reduce survival [22]. Heat stressed bed bugs may also use more energy and nutrients due to increased metabolism and sustained heat counter-measures [43, 63, 64]. Starved individuals can also suffer more from external stressors. This is seen in dehydrating ticks and mosquitos that experience substantial energetic costs, and individuals with limited energy reserves may be more susceptible to environmental stress [65–67]. Similarly, blood-deprived bed bugs are known to be more susceptible to neurotoxicants and desiccant dust [68–70]. Water loss, energy drainage and heat damage constitute crucial survival factors that might have contributed to the fecundity and offspring effects observed in this study. Access to blood is consequently an element to consider in terms of effect-magnitude and potential contribution in an IPM-strategy. In our study, survival of the starved nymphs was lower than that of the fed adults. Provision of blood close to the heat stress may reduce the detrimental effects because they become sufficiently hydrated and supplied with new energy, although a meal poses a brief physiological strain as body temperature dramatically increases when drinking blood from a warm-blooded host [51]. The stress from long lasting heat exposure or starvation may also be affected by body size. Adults and fifth instar nymphs are considered more resistant to external stressors than smaller nymphs due to the higher surface to volume ratios in smaller individuals [36], and the negative population impact observed in this study may be even more severe in natural populations where all developmental stages are present. Future studies will profit from comparing more stages of both blood-fed and unfed status.

The applied value of heat treatments below 40°C may at first seem limited, and the long time span to achieve relevant effects appears to be the major obstacle in many cases. However, there are tremendous variation in the management approaches across continents, countries and societal classes. Variation in the acceptance levels towards infestations, life-style and financial availabilities of residents as well as knowledge regarding bed bug ecology, physiology, behaviour and control among pest control technicians can determine the strategies in use. The composition of building types, vernacular architecture, legislation and government ruling may also regulate available approaches for pest control companies and consequently create strategy disparities across countries. The timing of initial treatment can also vary greatly from case to case, and there are many situations where 2 weeks or more will pass from the time an infestation is detected until control efforts are initiated. In Norway, the major social housing owner in Oslo takes an average of 15 days from bed bug detection to the first control attempt (Procurement officer–Boligbygg Oslo KF, Espen Roligheten, pers. comm.). If those who suffer from an infestation can temporarily afford to move out of infested rooms to avoid getting bitten, or if they can stay for free with friends or family, the addition of sublethal heat stress can be relevant to improve the efficacy of the consecutive control methods. To vacate a room with established bed bug populations is also the general practice undertaken by the Norwegian Hospitality Association [71]. This is an ethically sound strategy that reduces the risk of a guest being bitten, unintended infestations being brought to the guests’ homes and unconsented exposure to pesticides. Therefore, a simple solution to benefit from any sublethal heat effects can on some occasions be to turn up the heat in rooms. As a non-chemical method of an IPM strategy heat stress may support other efforts by weakening the bed bug population. Well-insulated bedrooms, typically found in the Nordic countries and other cold areas, can reach 37–38°C using three 1500 W electrical ovens and a small fan. This rather moderate energy use of 1500 KWh for a 14-day treatment makes out less than 5% of the average control cost in Norway and may consequently be a cheap addition to management. In many situations it will be challenging to reach such temperatures in all parts of a room, and additional preparations may be required to expose all individuals to detrimental temperatures.

Another potential use of sub-lethal heat stress is treatment of heat sensitive objects. Art objects, electronics, delicate clothing etc. that are vulnerable to conventional heat treatments [72–74], may utilize heat chambers holding temperatures as low as 38°C for extended periods.

It is important to emphasise that sublethal heat is not a stand-alone control option, but may be considered on a case-to-case basis as a contributor to improved elimination success. Given sufficient time, heat stress may kill bed bugs and eggs, but equally important is the ability to weaken the population before, during and after application of conventional measures as part of an IPM solution. Additionally, the ability to undergo repair during intermittent stress relief indicates a need for persistently elevated temperatures. To further improve the effect of sublethal heat, regular stimulation with CO_2_ to mimic the presence of the host may increase bed bug activity [3, 27, 75–78]. This will reduce quiescent time in aggregations to increase water loss [3], shorten time to reach critical levels of energy drainage [1, 3] and beneficially elevate contact time with other killing agents present in the room [27, 79]. In turn, increased activity due to heat stress, starvation or stimulation may increase the possibility of undesirable dispersal of bed bugs to new rooms, and countermeasures to seal off possible escape routes with desiccant dusts, silicone rubber or other sealants, should therefore be considered.

Combined effects are expected to occur from additive control efforts against bed bugs, as all may impact bed bug survival. The individual contribution from each control effort can be hard to determine, but additive effects can make strong impact for final eradication success. The potential additive aspects of sublethal thermal stress in an IPM management system surely warrant further studies. In particular, it must be investigated in a controlled field setting how heat stress can be combined with other efforts to achieve an improved management result.

## Supporting information

S1 Table Long-term effects of heat stress on *Cimex lectularius* nymphs. (DOCX)

S1 File Written consent donation of blood. (PDF)

S2 File Underlying data Rukke et al. (XLSX)
