# Supplementary material for: Temperature stress deteriorates bed bug (Cimex lectularius) populations through decreased survival, fecundity and offspring success
Source: PLoS One. 2018 Mar 14;13(3):e0193788. doi: 10.1371/journal.pone.0193788 (PMC5851602; doi:10.1371/journal.pone.0193788)
Supplement: S1 Table — (DOCX) [file pone.0193788.s001.docx]

| Source | *df* | SS | MS | *F* | *P* |  |  |  |  |
| --- | --- | --- | --- | --- | --- | --- | --- | --- | --- |
| Temp | 3 | 1.395 | 0.465 | 11.6 | <0.001 |  |  |  |  |
| Length | 1 | <0.001 | <0.001 | 0.0 | 0.931 |  |  |  |  |
| T × L | 3 | 0.105 | 0.035 | 0.9 | 0.468 |  |  |  |  |
| Residual | 32 | 1.285 | 0.040 |  |  |  |  |  |  |
| Total | 39 | 2.785 | 0.071 |  |  |  |  |  |  |
|  |  |  | | | | |  |  |  |

Eight week development of *Cimex lectularius* nymphs that hatched 7 weeks after the disrupted heat treatment of their parents. Constant treatments were too few to run a two-way analysis of variance on the effect of previous parental heat stress. Described are the effects of treatment length (Length), treatment temperature (Temp) and the interaction of these two variables.
