# Supplementary material for: Temperature stress deteriorates bed bug (Cimex lectularius) populations through decreased survival, fecundity and offspring success
Source: PLoS One. 2018 Mar 14;13(3):e0193788. doi: 10.1371/journal.pone.0193788 (PMC5851602; doi:10.1371/journal.pone.0193788)
Supplement: S1 File — (PDF) [file pone.0193788.s002.pdf]

Norwegian Institute of Public Health

Oslo. 28/11 - 2017

Written informed consent regarding donation of blood

Blood was not used as experimental samples, but as a food source for bed bug stock cultures. This is a part of a standardized institutional insect rearing regimen which do not require ethics approval according to the Regional Comities for Medical and Health Research Ethics – www.helseforskning.etikkom.no

Additionally, Bjørn Arne Rukke and Anders Aak, hereby declare that we have voluntarily donated 10 mL each 14 days of our blood to feed the bed bugs used in the experiments of the manuscript "Temperature stress deteriorates bed bug (*Cimex lectularius*) populations through increased mortality, reduced fecundity and maternal effects." Rukke and Aak are two of the authors of this manuscript.

Bjørn Arne Rukke	[signature]
Bjørn Arne Rukke	Anders Aak
